# Plasma Actin-free Gc-globulin in Patients with Chronic or Acute-on-chronic Liver Failure Caused by Hepatitis B Virus

**DOI:** 10.4021/gr2009.07.1300

**Published:** 2009-07-20

**Authors:** Huan Liu, Tao Han, Shi Xiang Xiao, Jun Li, Jun Lee, Yan Li, Zheng Yan Zhu, You Qing Xu

**Affiliations:** a Department of Hepatology, Tianjin Institute of Hepatobiliary Disease, Tianjin Key Laboratory of Artificial Cell, Tianjin Third Central Hospital, Tianjin Medical University, Tianjin 300170, China; b Department of Gastroenterology, Beijing Tiantan Hospital, Capital Medical University, Beijing, 100050, China; d These authors contributed equally to this work

**Keywords:** Gc globulin, Liver failure, Cirrhosis, Hepatitis B virus

## Abstract

**Background:**

Previous studies have confirmed that serum concentrations of actin-free Gc globulin (Af-Gc globulin) may provide prognostic information in patients withacute liver failure (ALF). However, until now the relation between plasma Af-Gc globulin levels and chronic or acute-on-chronic liver failure (CLF or ACLF) caused by HBV is unknown.

**Methods:**

Plasma Af-Gc globulin in 56 patients with liver failure, in 23 patients with compensated liver cirrhosis (CR), and in 25 healthy controls were measured using enzyme-linked immunosorbent assay (ELISA). Serum alanine aminotransferase (ALT), aspartate aminotransferase (AST), choline esterase (CHE), Albumin (ALB), total bilirubin (TBIL), palsma international normalized ratio of prothrombin time (INR), and platelet (PLT) were also detected. The Child-Pugh score was calculated for each patient on admission.

**Results:**

Plasma Af-Gc globulin levels in CLF, ACLF and CR were significantly lower than that of healthy controls (P < 0.001, respectively). The median (range) Af-Gc globulin level at admission for the liver failure (CLF or ACLF) was significantly reduced compared with that of CR group (P ≤ 0.001); additionally, there was significant difference between CLF and ACLF patients (P < 0.001). In liver failure cohort, plasma Af-Gc globulin was significantly positive correlated with ALB, ALT, AST and CHE (P was 0.001, 0.001, 0.001, < 0.001, respectively). Meanwhile, there was significantly negative correlation between plasma Af-Gc globulin and Child-Pugh score (P = 0.02). The level of Af-Gc globulin in ascites or hydrothorax-infected liver failure patients were markedly lower than that of non-infected (P = 0.015), the levels of Af-Gc in encephalopathy presence were lower than in encephalopathy absence. No significant difference of Af-Gc was noted between non-survivors and survivors during the follow-up period in liver failure patients.

**Conclusions:**

Plasma Af-Gc globulin levels in liver failure patients are significantly reduced compared with compensated liver cirrhosis patients and healthy controls, however, it might not be used in the prognosis of liver failure.

## Introduction

Liver failure is a severe end-stage liver disease, it always progress to multiple organ dysfunction (MOF) and is associated with high mortality [1-3]. Resent studies have illustrated the importance of microvascular dysfunction and microthrombosis in progressive MOF [4, 5]. Animal studies have identified polymerized actin as a potent cause of thrombotic microcirculatory dysfunction [6]. This ubiquitous intracellular protein is released in large quantities from necrotic human cells, including hepatocytes.

Gc globulin is a hepatically synthesized multifunctional protein, involved in scavenging of actin released from necrotic cells, binding and transporting vitamin D metabolites [7, 8] and nonspecific immune defense functions [9-11]. Gc-globulin may also act as a precursor of macrophage activating factor [12, 13]. Actin is a predominant intracellular protein in most cells including hepatocytes [14] and escapes from cells in large quantities following cell necrosis. In recent years, many researches have showed that the level of Gc globulin significantly reduced after severe liver injury, especially in acute or fulminant liver failure (FHF). Additionally, the levels of Af-Gc have a significant relationship with the prognosis of diseases [15-19].

However, the pathophysiological process of chronic or acute-on-chronic liver failure (CLF or ACLF) is different from that of acute liver failure (ALF), the levels of plasma Gc globulin in these patients are not clear. In this study, we aimed to test a new fast enzyme-linked immunosorbent assay (ELISA) for determining actin-free Gc-globulin(Af-Gc globulin), we intended to investigate the association between the reductions of Af-Gc globulin and liver dysfunction in patients with chronic or acute-on-chronic liver failure.

## Patients and methods

### Patients

Fifty-six patients with liver failure (44 males, 12 females; median age 52, ranging from 27 to 79 years), and 23 patients with compensated cirrhosis (20 males, 3 females; median age 54, ranging from 39 to 74 years) between October 2007 and April 2008 at Tianjin Third Central Hospital were recruited. The study was approved by Tianjin Third Central Hospital Ethics Committee.

The diagnosis of liver failure was made according to the Chinese Diagnostic and Treatment Guidelines for Liver Failure (2006) [20], and the diagnosis of compensated liver cirrhosis (CR) was made by typical ultrasound appearance and clinical features. In liver failure cohort, 26 were ascites or hydrothorax-infected patients, hepatic encephalopathy presented in 21 patients, gastrointestinal bleeding presented in 17 patients, there were 32 (57.14%) survivors and 24 (42.86%) non-survivors during 30 days follow-up. We divided the liver failure group into two subgroups, one was CLF, and the other was ACLF. Patients with ACLF were defined as those with an acute deterioration in liver function. All patients enrolled were infected with HBV. The healthy control group included 19 males and 6 females, median age 34 years, ranging from 24 to 62 years. Those patients with concurrent hepatocellular carcinoma, advanced cardiopulmonary disease, primary kidney disease and diabetes mellitus were excluded.

### Blood sample collection

All the blood samples were collected in the morning when patients were admitted to our hospital. Blood collected in heparin was spun at 3000 r/min for 15 minutes, and the plasma obtained was immediately stored at -80 °C until Af-Gc globulin measurement was performed.

### Collection of clinical data

The data of clinical investigations were collected when patients were referred to the hospital. Serum alanine aminotransferase (ALT), aspartate aminotransferase (AST), choline esterase (CHE), Albumin (ALB), total bilirubin (TBIL), palsma international normalized ratio of prothrombin time (INR), platelet (PLT) were detected. Meanwhile, Child-Pugh scores were calculated on admission.

### Measurement of plasma Af-Gc globulin

Af-Gc globulin levels were determined in triplicate by use of a sandwich enzyme-linked immunosorbent assay technique that used a standardized protocol (AntibodyShop A/S, Gentofte, Denmark). Inter- and intra-assay variation coefficients were less than 5%.

### Statistical analysis

Data were presented as mean (range). To identify differences between groups, ANOVA and Mann-Whitney U tests were used. Correlations were analyzed by Spearman rank test. A P value of less than 0.05 was considered statistical significant. All analysis was performed by the Statistical Program for Social Sciences (SPSS 13.0 for Windows).

## Results

### Clinical characteristics of the study population

The demographic data of the study populations were listed in Table 1. The nature of liver failure patients were as follows: 33 were CLF and 23 were ACLF. Twenty-three were enrolled in CR group. Twenty-five healthy volunteers served as controls. Etiology of every group except healthy control was HBV-infection.

**Table 1:** Clinical characteristics of the study populations, median (range)

Characteristic	CLF	ACLF	CR	Healthy
No.	33	23	23	25
Age (yr), median (range)	54 (35 – 79)	50 (27 – 74)	54 (39 – 74)	34 (24 – 62)
Gender (M:F)	23:10	21:2	20:3	19:6
Cause	HBV (33)	HBV (23)	HBV (23)	HBV (0)
AST (IU/L)	57 (20 – 459)	296 (24 – 1611)	44 (22 – 201)	
ALB (g/L)	25 (17 – 41)	28 (21 – 34)	34 (31 – 38)	
CHE (Units)	1346 (697 – 4034)	2251 (522 – 4168)	3457 (3243 – 4814)	
TBIL (µmol/L)	162 (63 – 460)	240 (95.7 – 918.4)	39 (19 – 80)	
INR	2.46 (1.91 – 5.01)	2.36 (1.89 – 5.67)	1.29 (1.03 – 1.84)	
PLT (10^9^/L)	44 (16 – 172)	70 (17 – 166)	88 (31 – 218)	
Child-Pugh score	13 (11 – 14)	12 (10 – 13)	8 (6 – 10)	

CLF, chronic liver failure; ACLF, acute on chronic liver failure; CR: cirrhosis

### Af-Gc globulin levels

**Figure 1:**
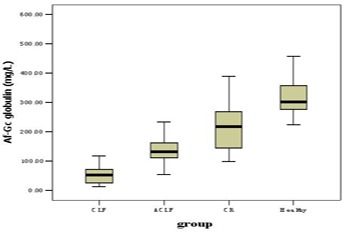
The levels of Af-Gc globulin in CLF, ACLF, CR and health controls

Af-Gc globulin levels for the groups of CLF, ACLF, CR and healthy controls were shown in Figure 1 and Table 2. Compared with the healthy controls, patients in CLF, ACLF and CR had significantly lower Af-Gc globulin levels, P < 0.001. The level of Af-Gc in liver failure (CLF or ACLF) was markedly reduced than that of CR (P ≤ 0.001). We also found a significant difference between CLF and ACLF (P < 0.001).

**Table 2:** Af-Gc globulin levels in each group

Group	No.	Gender (M:F)	Age (years)	Af-Gc globulin (mg/L)
CLF	33	23:10	54 (35 – 79)	52.45 (12.02 – 169.47) ^a, b, d^
ACLF	23	21:2	50 (27 – 74)	131.17 (53.73 – 374.80) ^a, c^
CR	23	20:3	54 (39 – 74)	218.40 (98.19 – 389.51) ^a^
Control	25	19:6	34 (24 – 62)	301.38 (223.72 – 520.53)

^a^P < 0.001 vs healthy; ^b^P < 0.001 vs CR;^c^P = 0.001 vs CR;^d^P < 0.001 vs ACLF. CLF, chronic liver failure; ACLF, acute on chronic liver failure; CR, cirrhosis

### Af-Gc globulin and clinical index in liver failure patients

Table 3 shows the relation between Af-Gc and other clinical index. There were strong positive relation between Af-Gc levels and ALB, CHE, ALT, AST (P = 0.001, < 0.001, < 0.001, < 0.001, respectively); Af-Gc globulin was correlated with Child-Pugh score in liver failure patients (r = – 0.313, P = 0.02).

**Table 3:** Correlation Coefficients (r) and P Values of laboratory test vs Af-Gc globulin in liver failure patients

Characteristic	r value	P value
ALB	0.422	0.001
CHE	0.539	< 0.001
ALT	0.428	0.001
AST	0.426	0.001
TBIL	0.245	0.069
INR	– 0.085	0.54
Child-Pugh score	– 0.313	0.02
PLT	0.228	0.097

### Af-Gc Globulin levels and complications of liver failure

Comparisons were examined in the liver failure patients (n = 56) to investigate the influence of complications to the levels of Af-Gc globulin (Table 4). The levels of Af-Gc globulin in ascites or hydrothorax-infected liver failure patients were markedly lower than that of non-infected (*P* = 0.015); No significant difference were noted in encephalopathy and GI bleeding (P = 0.083, 0.383, respectively). But a trend toward lower Af-Gc levels was seen in encephalopathy compared with non-encephalopathy in liver failure patients.

**Table 4:** Af-Gc levels and complications of liver failure, mean (range)

Characteristic	Present (mg/L)	Absent (mg/L)	P value
Encephalopathy	58.83 (12.06 – 374.8)	99.8 (12.02 – 331.67)	0.083
Infection	60.96 (12.02 – 233.14)	104.47 (32.65 – 374.80)	0.015
GI bleeding	66.28 (12.06 – 374.8)	90 (12.02 – 331.67)	0.383

### Af-Gc Globulin levels and prognosis

**Figure 2:**
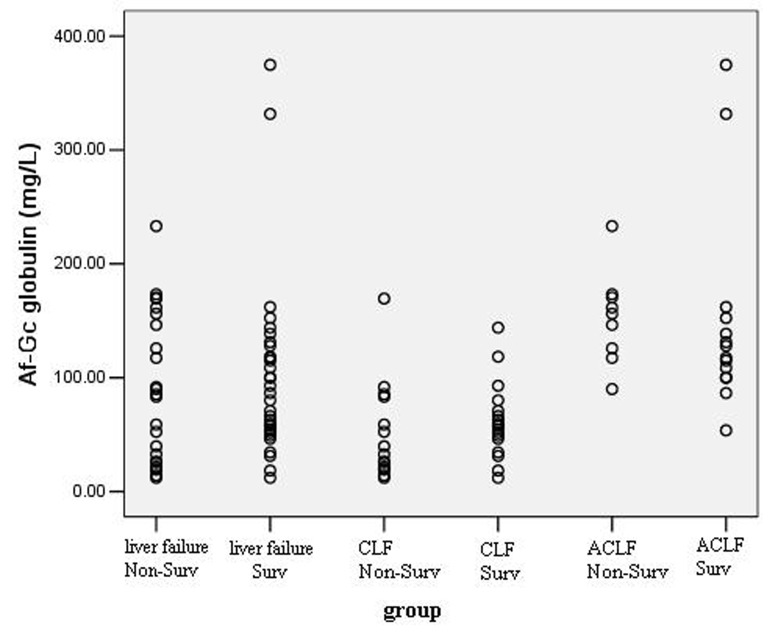
The levels of Af-Gc globulin in survivors and non-survivors in liver failure patients.

Of the 56 patients, the survival rate was 57.14% (32 patients) during 30 days follow-up. Figure 2 shows the distribution of Af-Gc globulin levels in survivors and non-survivors. There was no significant difference of Af-Gc levels between survivors and non-survivors in liver failure group. To further examine the Af-Gc Globulin for prognosis of liver failure, the liver failure patients were divided into two subgroups (CLF and ACLF), no significant difference of Af-Gc globulin in these two subgroups was seen (Table 5).

**Table 5:** Af-Gc levels for prognosis of liver failure, mean (range)

Group	No.	Non-survivors	Survivors	P value
LF	56	84.36 (12.06 – 233.14)	83.30 (12.02 – 374.80)	> 0.05
CLF	33	32.65 (12.06 – 169.47)	57.77 (12.02 – 143.94)	> 0.05
ACLF	23	156.18 (90.00 – 233.14)	122.71 (53.73 – 374.80)	> 0.05

CLF, chronic liver failure; ACLF, acute on chronic liver failure; CR, cirrhosis

## Discussion

Liver failure is viewed as a net loss of functioning hepatocytes to the point that surviving hepatocytes cannot maintain adequate metabolic functions. This represents either a failure to regenerate after partial hepatectomy or accelerated destruction of hepatocytes from necrosis or apoptosis [21]. According to the pathophysiological basis and progressive rate, liver failure can divided into acute liver failure (ALF), sub-acute liver failure (SALF), acute on chronic liver failure (ACLF) and chronic liver failure (CLF) [20]. In any condition, the lack of the metabolic and regulatory function of the liver results in life-threatening complications that may include bleeding, renal failure, hepatic encephalopathy or cerebral edema, cardiovascular failure, and susceptibility to infections culminating in multi-organ failure [22].

Gc globulin (also known as group-specific component, vitamin D-binding protein) belongs to the albumin superfamily of binding proteins, which includes Gc-globulin, albumin, α-fetoprotein, and afamin [23]. Apart from its specific sterol binding capacity, Gc globulin exerts several other important biological functions such as actin scavenging, fatty acid transport, macrophage activation and chemotaxis [24]. It is of note that, only a minority of 5% of total plasma Gc-globulin is occupied by vitamin D, leaving the majority free for scavenger functions [23]. To date, Gc-globulin has been recognized widely as a protein with markedly decreased concentrations in inflammatory and necrotic diseases. Previous studies including a large number of patients have confirmed that serum levels of the actin scavenger Gc-globulin are severely reduced in ALF or FHF by rapid clearance of Gc-globulin [25, 26]. Our study also demonstrates profound reductions in circulating Af-Gc globulin levels in CLF, ACLF and to a lesser extent in compensated liver cirrhosis by using a novel and rapid assay. These findings were in consistent with the results of Masuda et al [27]. The magnitude of reduction of this molecule is closely related to the degree of organ dysfunction and severity of the inflammatory response evoked after superimposed hepatic injury.

ACLF is defined as acute deterioration in liver function in a patient with preexisting chronic liver disease; CLF is different from ACLF, it develops into dysfunction gradually based on the liver cirrhosis. It is difficult to discriminate them in clinic. By this purpose, we compared the Af-Gc levels between CLF and ACLF, the result showed a statistically significant lower levels of Af-Gc in CLF than ACLF. For this, it can be seen that the measurement of Af-Gc may further help us to discriminate them.

ALB, ALT, AST, choline esterase (CHE) is highly expressed in hepatic cells or synthesized by hepatocytes, and their levels or activities can directly reflect the state of liver function. Our study found that the levels of Af-Gc globulin in liver failure patients had a significantly positive relationship with ALB, ALT, AST, CHE, that is to say, the level of Af-Gc can also be used to evaluate the degree of hepatic injury.

Child-Pugh score is the most widely-used liver functional assessment system for patients with liver disease. Our results showed Af-Gc globulin had a strong negative correlation with Child-Pugh score, so it can also be used as predictor of liver function. Antoniades et al [28] demonstrated the levels of Af-Gc globulin had a significantly positive correlation with PLT in unstable cirrhosis patients. Our study also verified this result. But there was somewhat difference from that of Antoniades [3], we found a weak positive relationship with TBIL, which is different from the previous study. The reasons may involve many aspects, for example, our study populations were CLF or ACLF patients who were infected by HBV, the Antoniades’s enrolled population was ALF that were mostly induced by acetaminophen. Therefore, it is necessary to further investigate the relationship between them in larger series liver failure patients caused by hepatitis B virus in the future.

Ascites, spontaneous bacterial peritonitis, hepatic encephalopathy, variceal bleeding and other serious complications will happen when the liver diseases progress to end-stage. In our study, we identified that some complications could influence the levels of Af-Gc globulin in liver failure patients. For example, we found that the Af-Gc levels were significantly lower in the ascites or hydrothorax-infected patients (60.96 mg/L) than that in the non-infected patients (104.47 mg/L). It was reported that the response of Gc-globulin involve in nonspecific immune defense function and it is markedly decreased in inflammatory [15]. The above phenomenon also suggest that reduced Gc-globulin levels must therefore reflect an increased Gc-globulin consumption, either because of actin complexing and removal from the circulation or because of consumption as part of other immune-related functions [29, 30]. Additionally, as we demonstrated, within the liver failure cohort, there was no difference in Af-Gc levels between presence and absence of hepatic encephalopathy, but it had a trend that with the hepatic encephalopathy, the levels of Af-Gc would be lower than non-complicated. This was in agreement with the previous reported results [19]. Moreover, we did not detect a difference between presence and absence of gastrointestinal bleeding.

The distribution of Af-Gc levels in survivors and non-survivors during the follow-up period showed there was no significant difference between them in liver failure cohort. To better validate our finding, we chose to divide the liver failure patients into 2 subgroups (CLF and ACLF) on the basis of Diagnostic and treatment guidelines for liver failure in 2006 [20]. The results in the two subgroups were almost identical to that of liver failure group. That is to say, the level of Af-Gc globulin was not an independent predictor of mortality in liver failure patients, despite it mirrored hepatic dysfunction. This was in agreement with the reported results [28, 31, 32]. It is of note that many intra or extrahepatic complications may influence the prognosis of liver failure patients. Possibly, according to previous researches, combining Af-Gc globulin with other known prognostic markers, including factor V levels [33], alfa-fetoprotein [34], or phosphate [35], may prove more accurate, and such analyses are underway from our group.

In conclusion, to some extent, the measurement of plasma Af-Gc globulin can reflect the degree of hepatocellular necrosis, and it can be used to discriminate liver failure and non liver failure patients. Whereas the prognosis of liver failure patients were affected by many factors, and our data do not favor the levels of Af-Gc globulin as an independent predictor of mortality in liver failure patients (CLF and ACLF).The next step we can combine with other known prognostic markers to evaluate its role in the prognosis of liver failure. It is likely that deficiency of this protein plays an important role in the pathophysiology of liver failure and should be further studied in the future.
